# MRI in axial spondyloarthritis: understanding an ‘ASAS-positive MRI’ and the ASAS classification criteria

**DOI:** 10.1007/s00256-022-04018-4

**Published:** 2022-02-23

**Authors:** Torsten Diekhoff, Robert Lambert, Kay Geert Hermann

**Affiliations:** 1 Department of Radiology (CCM), Charité - Universitätsmedizin Berlin, Campus Mitte, Humboldt-Universität Zu Berlin, Freie Universität Berlin, Charitéplatz 1, 10117 Berlin, Germany; 2 Department of Radiology & Diagnostic Imaging, University of Alberta, 2A2.41MC, 8440 - 112 Street, Edmonton, AB T6G 2B7 Canada; 3 Medical Imaging Consultants, 202-11010 - 101 Street, Edmonton, AB T5H 4B9 Canada

**Keywords:** Axial spondyloarthritis, Magnetic resonance imaging, Classification

## Abstract

In 2009, the Assessment of SpondyloArthritis international Society (ASAS) published a definition of ‘active sacroiliitis on magnetic resonance imaging (MRI) for classification of axial spondyloarthritis’. This new definition of an ‘ASAS-positive MRI’ was integral to new classification criteria for axial spondyloarthritis that were published in the same year. The ASAS MRI definition had the considerable advantage of simplicity and the definition gained popularity as guidance for interpreting MRI of the sacroiliac joints in clinical practice. However, classification criteria are not designed for use in clinical practice with the consequence that overreliance on the presence of bone marrow edema, which is the principal determinant of an ‘ASAS-positive MRI’, may result in a tendency to overcall inflammatory sacroiliitis in the clinical setting. This article aims to inform the reader about the rationale behind the ASAS definition of a positive MRI and ASAS classification criteria, their proper use in research and why they should not be used in clinical practice. The article also contains guidance for an updated imaging protocol and interpretation of images including typical imaging findings, differential diagnosis, and common pitfalls.

## Introduction

Axial spondyloarthritis (axSpA) is a complex group of disorders that is clinically characterized by inflammatory back pain, affection of younger, predominantly male patients, an association with the human leukocyte antigen B27 (HLA-B27) and inflammation of the spine and sacroiliac joints (SIJ) [1]. This group of disorders includes Ankylosing Spondylitis (AS) and other inflammatory arthropathies when they involve the axial skeleton. Early diagnosis is important because spinal involvement can be disabling and disease-modifying treatment may prevent long-term loss of function and late-stage complications [2, 3]. While defining the population of patients that suffers from axSpA is complex, there is universal agreement that imaging of the axial skeleton is a crucial component of diagnostic ascertainment in axSpA.

The objective detection of disease is more often critical for diagnosis in axSpA than peripheral arthritis as the spine is much less amenable to clinical examination than peripheral joints, and laboratory tests are non-specific for axSpA [4, 5]. Historically, radiography has been used to detect sacroiliitis and ankylosis and the modified New-York-Criteria (mNYC) were developed for the classification of patients with structural damage in the sacroiliac joints (SIJ) [6]. Although radiography cannot detect the initial inflammatory stage that precedes structural damage, in the past this was less important because no disease-modifying therapies were available for AS. About thirty years ago, new biological therapies were developed and shown to be effective in the early stages of peripheral arthritis. Despite this evidence, clinical trials were not easily performed in ‘early’ axSpA because classification criteria did not include a definition for axSpA prior to the development of radiographically evident structural damage. Around the same time, magnetic resonance imaging (MRI) revealed hitherto undetectable bone marrow inflammation. Now, for the first time, effective treatments were available and early disease could be detected, so the Assessment of SpondyloArthritis international Society (ASAS) developed new classification criteria, incorporating active inflammation of the SIJ on MRI, designed for research and therapy [7]. Since then, the definition of an ASAS-positive MRI has often been applied in clinical practice even though the definition was not designed for that purpose, resulting in a tendency to over diagnose sacroiliitis when only minor MRI findings were present [8, 9].

This article aims to explain the rationale behind the ASAS definition of a positive MRI and ASAS classification criteria, their proper use in research and why they should not be used in clinical practice. The article also contains guidance for an updated imaging protocol and interpretation of images.

## Imaging of the sacroiliac joints in axSpA

MRI of the SIJ can show active inflammation in the form of osteitis (bone marrow edema (BME)), enthesitis, and capsulitis. Of these, osteitis is the most important observation for the diagnosis of axSpA with enthesitis and capsulitis being more useful as supporting evidence when the pattern of osteitis is indeterminate [10]. Osteitis is typically found in the bone marrow adjacent to the cartilaginous joint surface and has high signal in fat-suppressed T2-weighted or T1-weighted post-contrast sequences, usually with some diminished T1w-signal [11]. Articular surface erosion occurs early in sacroiliitis but is often quite subtle in the early stages [12]. As structural damage progresses, erosion and sclerosis become more visible. When the active inflammatory phase subsides, repair processes take over and the main MRI observations include (a) fat lesions (bright T1w-signal) that replace the previously inflamed non-eroded bone marrow [13], (b) new bone formation that appears as fat signal within areas of erosion (commonly called backfill, also bright T1w-signal) [14], and (c) progressive new bone formation that crosses the original joint space, initially as bone budding and eventually as ankylosis [15]. On MRI, many of the structural damage lesions are best seen on T1-weighted spin echo sequences. Radiography of the SIJ is useful for detecting the more advanced stages of sacroiliitis demonstrating erosion associated with joint space widening and narrowing, and ankylosis. Sclerosis is also quite well seen but tends to be non-specific. CT is the gold standard for detecting most structural damage changes, apart from fat lesions [16].

## ASAS classification criteria

The ASAS classification criteria for axSpA have entry criteria for evaluation of a patient that must be fulfilled before any other tests or filters are applied. These initial criteria are three or more months of continuous back pain and an onset of back pain at an age younger than 45 years. Following this initial step, the ASAS classification criteria have a clinical and an imaging arm [7]. For the fulfilment of the clinical arm, there is no need for any findings on any imaging. HLA-B27 positivity and two clinical features (e.g. peripheral enthesitis) are sufficient to classify the cause of the back pain as axSpA. To fulfil the imaging arm, either structural damage positivity according to the radiographic mNYC or active inflammation as evidenced by an ASAS-positive MRI, plus one clinical feature, is required. Both sensitivity and specificity of the ASAS criteria have been reported to be near 80% when applied to the appropriate population [7, 17]. However, it should be noted that the reported level of accuracy of the criteria is dependent on two additional filters that have been applied in most publications but are often not emphasized—(a) the ascertainment of meeting the criteria is made by experts in the field (rheumatologists that publish research) and, (b) in most cases, the at-risk population was filtered before referral to a rheumatologist by a primary care physician because of a concern for a diagnosis of inflammatory arthropathy. This is discussed further below.

## The definition of an ASAS-positive MRI

The ASAS definition of active sacroiliitis on MRI (an ASAS-positive MRI) requires the presence of BME “in a typical anatomical area (subchondral bone)” and the “MRI appearance must be highly suggestive of axSpA” [18, 19]. Other inflammatory lesions, such as enthesitis or capsulitis, and the sole presence of structural lesions do not meet the definition of “active sacroiliitis on MRI”. The final component of the original 2009 definition included the requirement for “bone marrow edema on at least two consecutive slices or at more than one location in a single slice” [19]. However, this quantitative component (i.e., two slices or two locations) proved to be a major problem in the practical application of the definition. By unanimous consensus, ASAS updated the definition of a positive MRI in 2016 removing quantitation from the required elements of the definition and inserting it into “Guidelines for the application of the Definition” [18]. The requirement of BME in two slices or locations in the 2009 definition has subsequently been discredited by ASAS and other sources [20].

**Fig. 1:**
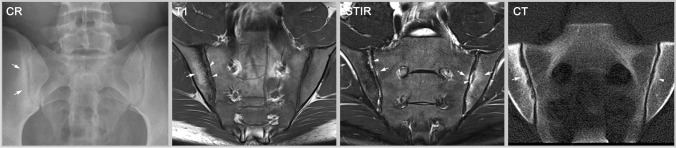
Sacroiliitis—findings typical for axSpA. The radiography (CR) shows sclerosis and erosions (arrows) in the right SIJ whereas the left SIJ appears normal. T1w-MRI additionally reveals fatty marrow metaplasia (arrow) and erosion (arrowheads) in the right SIJ; and STIR displays inflammatory bone marrow lesions (arrows) bilaterally, especially in the left SIJ, as well as inflammation within the joint space (arrowheads) bilaterally. Computed tomography (CT) depicts erosions and sclerosis with high resolution (arrow) but cannot detect active bone marrow inflammation. In the left SIJ, CT shows only a slight erosion of the iliac cortical surface and mild sclerosis (arrowhead). All three modalities are typical for sacroiliitis caused by axial spondyloarthritis (axSpA)

There has been much debate about the definition of an ASAS-positive MRI [21]. The rationale for the definition is multifactorial. (A) Early axSpA is characterized by active inflammation of the axial skeleton, and, when this is detectable with imaging, the SIJ are involved in over 95% of patients (less than 5% show active inflammation restricted to the spine). Therefore, the spine is not a focus for the definition [22]. (B) Structural lesions are generally well-depicted on X-ray, for which established criteria exist [6]. (C) The accuracy of MRI for detection of structural lesions was unclear at the time the criteria were developed. (D) Osteitis of the SIJ is the most predictive finding of early, non-radiographic axSpA and represents the situation in which a patient with early disease is most likely to progress to develop AS and need biologic therapy [23, 24]. (E) Small, focal spots of edema or edema-like lesions are often non-specific or artifacts [25, 26]. Therefore, a minimum amount of osteitis must be present. (F) Finally, the definition recognized the importance of the global perspective of an interpretation that the total constellation of observations—including the distribution of changes and other findings (especially structural lesions in the SIJ)—must be ‘highly suggestive’ of the presence of inflammatory sacroiliitis [18]. In summary, inflammatory changes in the SIJ are required and should be adjacent to the cartilaginous surface of the joint (not the ligamentous part) with a sufficient amount of BME that is not explained by artifact or other causes [15]; the distribution should be indicative of axSpA, not restricted or pronounced in the most ventral portion of the joint [27]; structural lesions must be considered, e.g. the presence of erosions or fat lesions would support the impression of axSpA [28]; and the constellation of findings must be “highly suggestive” of axSpA (see also Fig. 1). Only, when these conditions are sufficiently met can the MRI be designated as ‘ASAS-positive’. It must be emphasized that “bone marrow edema on two consecutive MRI slices” alone is not, and never was, sufficient to meet the definition of an ASAS-positive MRI.

## Clinical diagnosis vs classification

The ASAS classification criteria cannot be applied for establishing the diagnosis of axSpA in clinical practice. Diagnostic criteria are not well-designed if approximately 20% of subjects fulfilling the criteria do not suffer from the disease and the same share of patients with the disease do not fulfil the criteria. Even in ideal circumstances, the sensitivity and specificity are reported to be about 80%, but why do the criteria perform poorly for diagnosis and what were they designed for?

The diagnostic process in clinical practice is focused on a single person and is characterized by a combination of a weighted consideration of all aspects of the patient history, plus positive and negative test results and the exclusion of possible differential diagnoses [29]. The ultimate aim is to establish a diagnosis in an individual person; however, the final result is always a probability that the patient suffers from the respective disease [30]. The diagnosis will often be re-evaluated based on response or lack of response to therapy or during follow-up when the weighted consideration of findings may change with altered symptoms or new information [31].

Classification is focused on groups, not individuals, and the aim of classification is to create a homogenous collective of patients for research purposes that may be used to test a new therapy or diagnostic method. When research subjects are divided into test and control groups, it is essential that the two groups are the same with the same overall disease severity and likelihood that they have the disease under investigation. Usually, when patients are being recruited for a research project, as soon as enough positive findings are present to fulfil the entry criteria, the patient enters the trial. However, classification criteria do not generally consider negative test results. The conclusion is a yes-or-no answer with no consideration of probabilities for the individual subject. Therefore, classification criteria are designed to be applied only in patients with an established diagnosis and they are not designed to establish the likelihood of diagnosis or to be used for diagnostic purposes [32].

**Fig. 2:**
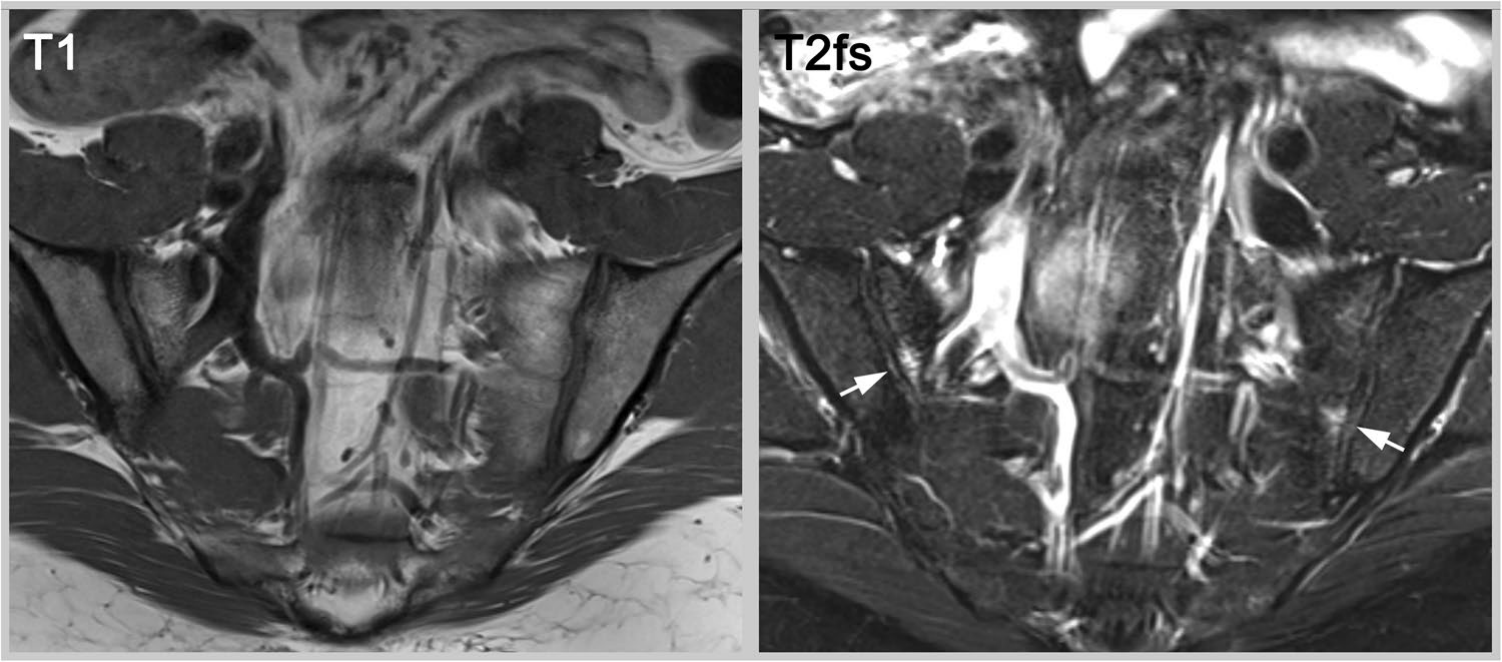
Bone marrow edema in two locations in an asymptomatic female volunteer. The T2w fat saturated (T2fs) sequence shows small bilateral bone marrow lesions (arrows) in an asymptomatic female. Whereas the MRI meets the outdated, quantitative definition of sufficient bone marrow edema for an ASAS-positive MRI, diagnosis is not supported by any structural lesions, and the individual would not fulfill the entry criteria of the ASAS classification system

A common example would be a marathon runner with enthesitis of the Achilles tendon who also happens to have some stress-related bone marrow edema at the SIJ in MRI [33, 34]. This patient would fulfil the ASAS classification criteria, whereas the probability for an axSpA diagnosis might be quite small. Bone marrow edema and enthesitis can both be explained by increased mechanical stress (consideration of alternative diagnoses) and if the patient is HLA-B27 negative, has normal CRP and no structural imaging findings (consideration of negative test results), then SpA would be highly unlikely [35]. Furthermore, enthesitis might be less predictive compared to other SpA-features such as a positive family history or psoriasis (weighting of the findings) [36], see also Fig. 2.

**Fig. 3:**
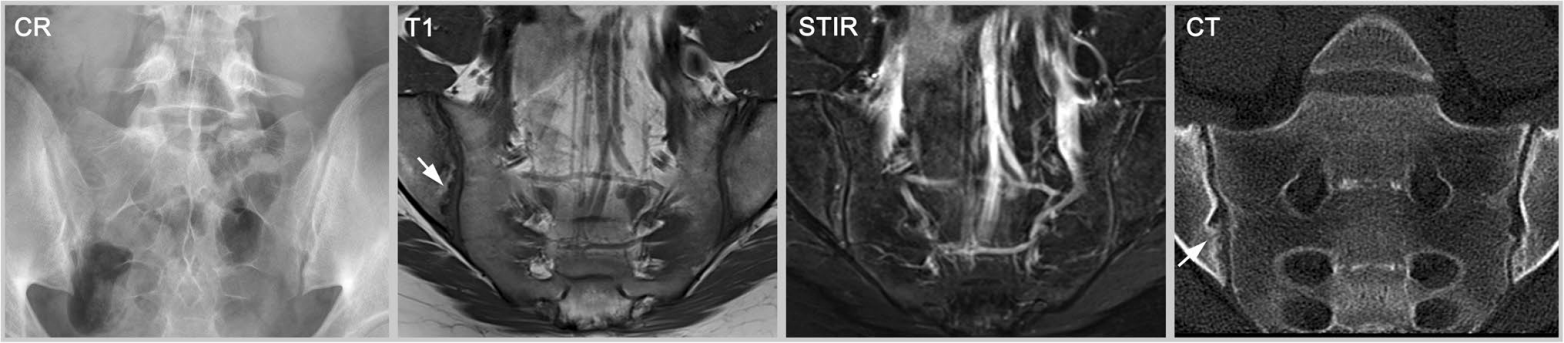
Early axSpA without active inflammation. This 24-year-old male patient has negative radiography but unequivocal erosions in MRI-T1 and CT (arrows). However, he shows no osteitis on STIR. While he does not fulfill the definition of a positive imaging test (radiography is negative and MRI shows no active inflammation), the erosions still support a clinical diagnosis of axSpA

**Fig. 4:**
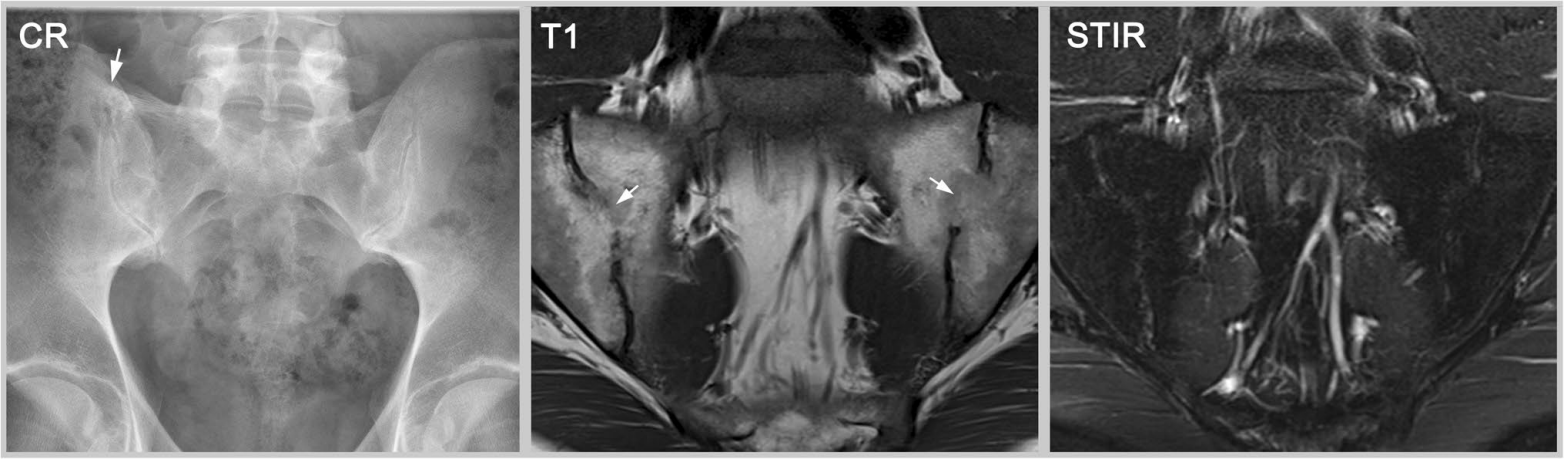
Established sacroiliitis not fulfilling the ASAS definition of a positive MRI. Despite radiography that only shows some capsule calcification (arrow), MRI-T1 clearly displays bilateral ankylosis in the midpart of the joint. The ankylosis is not amenable to detection by radiography because this part of the joint is not in parallel orientation to the X-ray beam. MRI-STIR shows no osteitis. This patient with established axSpA fulfills neither the radiographic standard (mNYC is negative) nor the ASAS definition for a positive MRI (no bone marrow edema) and represents another example of ‘false-negative’ imaging if the ASAS definitions are used for clinical diagnosis

On the other hand, a patient with confirmed diagnosis of axSpA might not fulfil the criteria because even though being HLA-B27 positive and having inflammatory back pain, the patient has no other SpA features, only few structural lesions and currently no inflammatory activity on imaging (see Figs. 3 and 4). In this case, the patient should be treated (the clinical diagnosis of axSpA is established) but not included in a clinical trial (the classification criteria are not fulfilled). Also, patients not meeting the entry criteria of symptom-onset before age 45 or shorter disease duration would be in this category [37].

## ASAS classification criteria in radiology science

In the same way as the presence of BME in fluid-sensitive sequences alone is not sufficient to state MRI positivity, MRI positivity itself is not enough to fulfil the classification criteria and being positively classified is not the same as having the diagnosis. Classification criteria are generally designed to be applied prospectively. Of course, it is quite feasible to apply these criteria retrospectively; however, in retrospective studies, data is often missing and outcomes may differ according to how or the order in which the filters are applied. Imagine you want to perform a retrospective study of SIJ MRIs to assess whether axSpA patients have less subcutaneous fat compared to non-axSpA patients. You might want to separate the patients into two groups, patients and controls, by using ASAS MRI positivity alone, but that approach does not reflect a proper application of the criteria: first, the entry criteria for classification (age and disease duration) are not considered; second, further criteria (HLA-B27 or clinical SpA features) are not considered; third, the actual diagnosis is not considered. The correct use would be to gather clinical information about the patients and separate both groups by their clinical records. Thereafter, exclude all axSpA-patients that do not fulfil the ASAS criteria by considering radiographic and MRI positivity for the imaging arm and then perform the analysis.

## “ASAS definition of a positive MRI” in clinical radiology care

Even if the ASAS definition were to be applied in clinical practice, it is inappropriate to condense the definition down to the simplest possible binary result—“a sufficient amount of bone marrow inflammation in two consecutive slices—Yes/No?”. The depiction of the SIJ with only a single coronal short-tau inversion recovery (STIR) sequence to screen for SIJ inflammation is inadequate for diagnostic purposes and is likely to identify many cases that have mild BME in the SIJ for other reasons. It is questionable as to whether the SIJ should ever be screened by MRI for sacroiliitis, and even if this were to be done, there is no evidence to support the use of the ASAS definition to determine ‘screen test positivity’. Diagnostic MRI of inflammatory sacroiliitis and its differential diagnoses needs a careful assessment of structural lesions and the same standards as for other anatomical regions should also apply to the SIJ [38]. To establish a clinical diagnosis, the observations of osteitis and structural damage of the SIJ must be interpreted in the context of the clinical setting. Only the person with all clinically relevant information can judge whether axSpA is truly present and that is more often the attending rheumatologist. In some circumstances, it is possible that the radiologist might be the person that gathers the last piece of essential information and could be certain about the final clinical diagnosis. However, it is rare for the radiologist to have all this information and, unfortunately, quite common that limited information is provided to the radiologist. Therefore, the radiologist should focus his interpretation on the likelihood that MRI findings are due to inflammatory sacroiliitis and should not be constrained in any way by a research definition created for the purposes of classification.

## Should I report ASAS positivity in clinical MRI?

It is a matter of intense debate as to whether a radiologist should record definitions, based on classification criteria, in a diagnostic clinical report and there are multiple reasons for not doing so. First, the ASAS MRI definition can only be applied if the patient meets the fundamental entry criteria for classification—continuous back pain for at least months and onset prior to age 45—and often the radiologist will be unaware as to whether the patient is eligible for the definition to be applied. Second, many radiologists are not fully aware of the details of the ASAS definition or changes to the definition. Third, stating that an MRI scan is ‘ASAS MRI negative’ may be very misleading. For example, a patient with no SIJ inflammation but with structural damage in the SIJ and florid inflammation in the spine and entheses due to axSpA is ‘ASAS MRI negative’ but may still meet ASAS classification criteria if an X-ray of SIJ was performed because the radiograph might be mNYc positive. Fourth, if the referring physician was not fully aware of the abovedescribed differences between classification and diagnosis, recording the MRI scan as ASAS-positive or ASAS-negative might be interpreted as a diagnostic statement. Fifth, in some countries, the indication for prescribing certain drugs depends on ASAS or mNYC positivity [39]. Legal problems could occur if, for example, an insurance provider links the approval of a certain therapy to classification and the radiology report states a different opinion from the conclusion of the rheumatologist [40]. Therefore, it is the opinion of the authors that clinical radiologists should focus on diagnostic ascertainment of the MRI and ‘ASAS MRI positivity’ should not be reported unless the answer to this question is specifically requested by the referring clinician. Many clinicians, such as most orthopaedic surgeons and general physicians, will not know how to use this information and including this statement in the report could be misleading. However, if the findings on MRI are consistent with sacroiliitis due to axSpA, it may be helpful for rheumatologists if the report clearly states in the conclusion whether ‘active inflammation’ is present or not.

## An SIJ MRI acquisition protocol for diagnosis

Some authors have proposed a minimum MRI protocol for the SIJ of an oblique coronal T1 and STIR sequence with sufficiently high resolution, before suggesting the diagnosis of axSpA [41]. However, this consensus is already out of date with increasing recognition by many radiologists that the minimum standard for an MRI protocol of the SIJ for diagnostic purposes should include at least 4 sequences: 3 in the semicoronal plane—(1) a T1-weighted sequence sensitive for marrow fat signal (such as T1 spin echo), (2) a T2-weighted water-sensitive sequence for BME (such as STIR or equivalent), (3) a sequence designed for optimal depiction of the bone-cartilage interface (articular surface) to allow improved visibility of erosion [12, 42, 43]; plus 1 sequence in the semiaxial plane that is orthogonal to the semicoronal sequences. This latter sequence has been shown to greatly assist in the ascertainment of BME patterns [44], and using only the coronal sequences will reveal patterns of BME that may appear to meet the 2009 ASAS definition of active sacroiliitis in 30–41% of athletes [26]. A 4-sequence protocol for MRI of the SIJ has been recommended by the radiologists of the European Society of Skeletal Radiology (ESSR) Arthritis Subcommittee since 2015 (“obligatory minimum: (1) coronal oblique T1-weighted, (2) coronal oblique STIR, (3) a cartilage sequence, and (4) visualization in two perpendicular planes”) and was endorsed by the MRI protocol working group of ASAS in January 2022 [45].

## Differential diagnosis of BME at the sacroiliac joint

There are certain conditions in which classification criteria fail. Multiple diseases in the SIJ can present with edema-like signal changes and mimic axSpA on MRI and clinical presentation [46–48]. As always, the more different and pronounced the findings are, the more certain is the judgement of the interpreter [20, 49].

One of the more common differential diagnoses that we encounter in clinical practice that can show variable degrees of bone marrow edema is osteitis condensans ilii, a stress- and often pregnancy-related condition with focus on the ventrocaudal aspect of the joint [50]. It typically occurs in young women after giving childbirth but can also appear in patients with obesity or a high amount of physical stress to the joint (e.g. riding or martial arts) [51, 52]. MRI can show variable stages of osteitis, fatty marrow metaplasia or sclerosis, but characteristically no or only few erosions [53]. In contrast, axSpA often shows erosion, backfill or ankylosis and the edema is more randomly distributed over the joint, without clear focus on the most antero-inferior part.

**Fig. 5:**
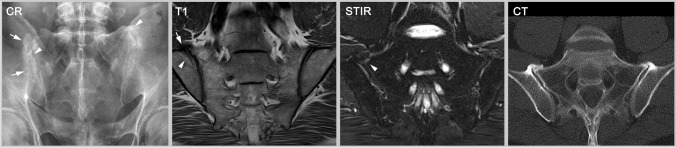
Fifty-six-year-old patient with osteoarthritis. Radiography can easily be falsely judged as positive because osteophytes can mimic erosion and sclerosis (arrows) and even partial ankylosis (arrowheads) when bone proliferation is superimposed over the joint but is in fact external to the articular surface and is around the joint. On MRI, it may be harder to see the bridging osteophytes (arrow), especially compared to CT. Also, osteoarthritis can show small bone marrow lesions with fat deposition (arrowhead in T1) or edema (arrowhead in STIR), especially near osteophytes. In this case, the radiography is ‘false-positive’ but the MRI is ‘true-negative’ for sacroiliitis

Other degenerative findings and osteoarthritis are less common in the age group most at risk for disease onset but become increasingly common in middle-aged and older patients [54]. Usually, the bone marrow changes are small foci of BME in the cartilaginous part of the joint. One or two small erosions or minor irregularity of the articular surface may be seen with subchondral cysts, which may be gas-filled, and osteophytes may be seen anywhere around the perimeter of the joint (see Fig. 5). Osteoarthritis and related changes are especially common in patients with scoliosis or developmental anomalies such as transitional vertebrae or accessory joint facets [55]. Although patients with osteoarthritis can show subchondral osteitis anywhere in the joint or close to osteophytes, the small foci of bright signal are usually less than 1 cm in diameter and are frequently seen on only 1 or 2 slices. In addition, physiological regression of erythropoietic marrow with increasing age may mimic fat metaplasia seen in bone marrow in SpA contributing to the difficulty in distinguishing OA from SpA in the older subject [56].

**Fig. 6:**
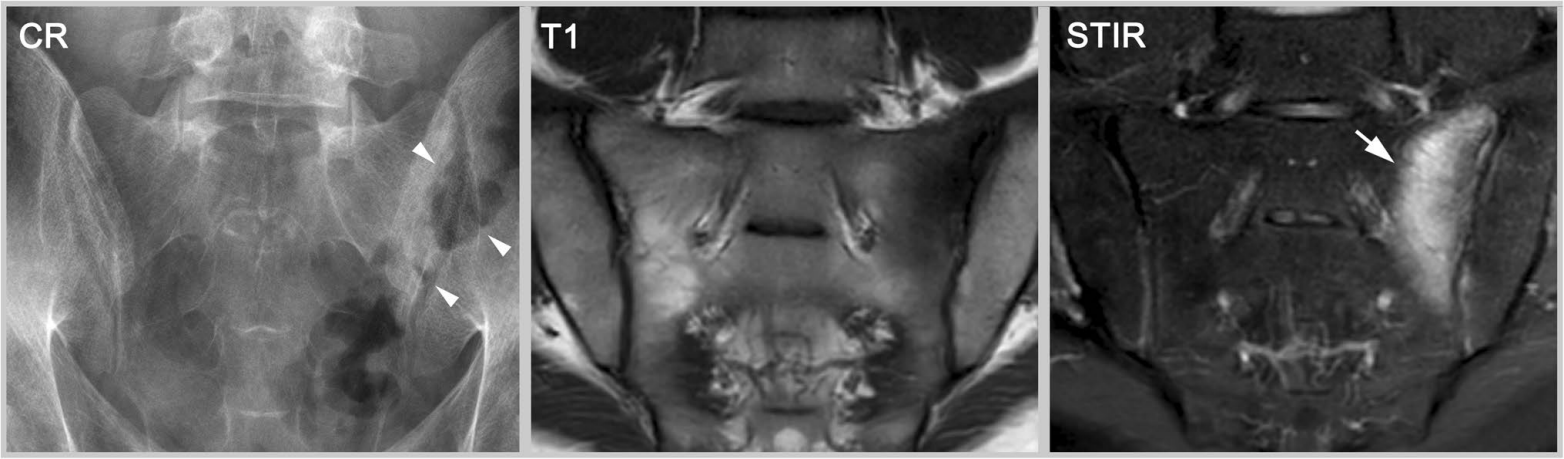
Fatigue fracture of the left sacrum in a patient with extensive sporting activities. On radiography it can be hard to distinguish erosion from superposition of bowel gas (arrow heads). Cross-sectional imaging shows bone marrow edema (arrow) but no typical erosions or fat lesions. The distribution of imaging findings (unilaterally and in the sacrum) and the patient’s history (extensive horse riding) point towards a more mechanical genesis—a fatigue fracture

Traumatic or insufficiency fractures of the sacrum cause bone marrow edema and can occasionally appear to cause focal articular surface erosion. Here the depiction of the fracture line or the distribution of the findings can help to distinguish these from axSpA which typically involves the iliac bone more than the sacrum due to the relatively thin cartilage surface (see Fig. 6) [57].

**Fig. 7:**
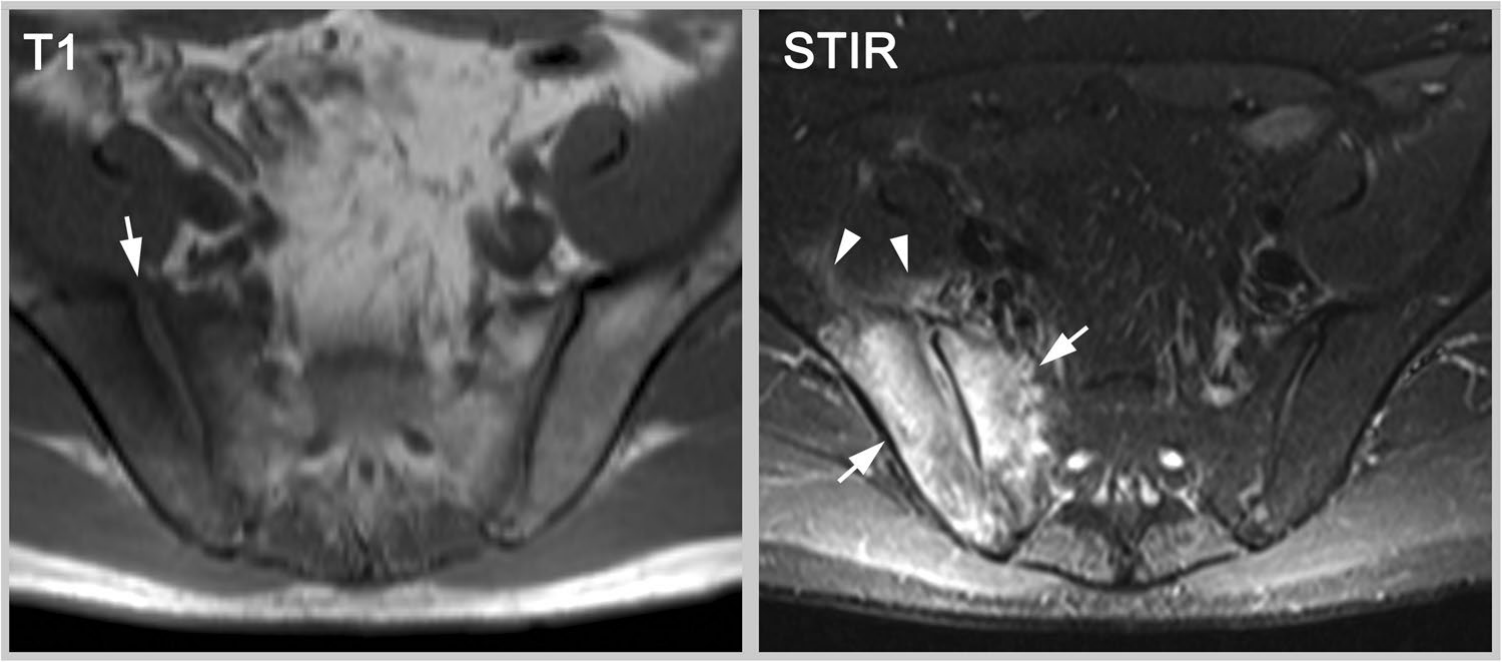
Septic sacroiliitis. MRI-T1 shows widening of the joint space from erosion (arrow) and MRI-STIR depicts severe bone marrow edema (arrows) and soft-tissue reaction (arrowheads). The extent of bone marrow edema is uncommon and the extent of soft-tissue inflammation is rare in axial spondyloarthritis, plus other structural lesions, such as fat metaplasia or backfill, are absent. Notably, there is also no lesion in the left SIJ

Occasionally, septic sacroiliitis can be difficult to differentiate from sacroiliitis in axSpA, especially in early forms [58]. However, the patients usually differ in their clinical presentation. Typical findings of reparation that are characteristic for axSpA (fat metaplasia, backfill, bone budding and focal ankylosis) are rare in septic arthritis and ankylosis occurs only after resolution of infection (see Fig. 7). Other systemic diseases can also manifest themselves in the SIJ [59]. Gouty arthritis of the axial skeleton is sometimes best seen with CT and the observer must consider age, gender, nutrition, and other factors which will profoundly influence the likelihood of this diagnosis [60].

## Conclusion

Classification criteria in rheumatology are designed to generate a homogeneous patient collective for clinical trials and other research purposes. They should be applied only when a diagnosis has already been established and they are neither diagnostic criteria nor designed as a replacement when no diagnostic criteria exist. The definition of a positive imaging study, that forms an integral part of classification criteria, may be seen as an expert consensus to create reasonable sensitivity and specificity for a diagnostic test when used for research purposes. Such definitions provide limited guidance for image interpretation in daily practice, should not be relied upon for diagnostic ascertainment, and should not be recorded in MRI reports, unless specifically requested by the referring physician.
